# Supplementary material for: Comparison of Microbial Diversity of Two Typical Volcanic Soils in Wudalianchi, China
Source: Microorganisms. 2024 Mar 26;12(4):656. doi: 10.3390/microorganisms12040656 (PMC11051941; doi:10.3390/microorganisms12040656)
Supplement: Supplementary file 1 [file microorganisms-12-00656-s001.zip › microorganisms-2898233-supplementary.pdf]

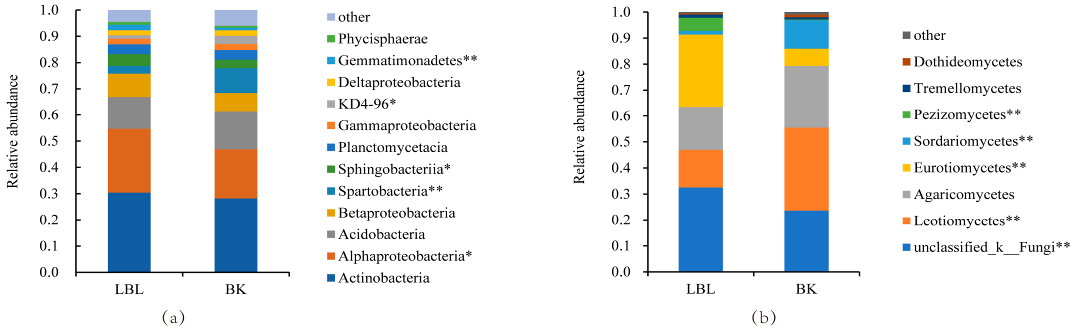

Figure S1. Bacterial (a) and fungal (b) community composition at the class. The class with a relative abundance of < 0.1% in soils were presented in other class". (** $p < 0.01$) and (* $p < 0.05$) indicate significant, differences between abundances in LBL and BK soils based on Wilcoxon signed-rank.

At the class level of bacterial community, the abundances of Gemmatimonadetes, Sphingobacteriia and Alphaproteobacteria were significantly higher in LBL than those in BK (Figure S1a). the abundances of KD4-96 and Spartobacteria were significantly lower in LBL than those in BK (Figure S1a). At the class level of fungal community, the abundances of Eurotiomycetes and Pezizomycetes were significantly higher in LBL than those in BK (Figure S1b). the abundances of Leotiomycetes and Sordariomycetes were significantly lower in LBL than those in BK (Figure S1b).

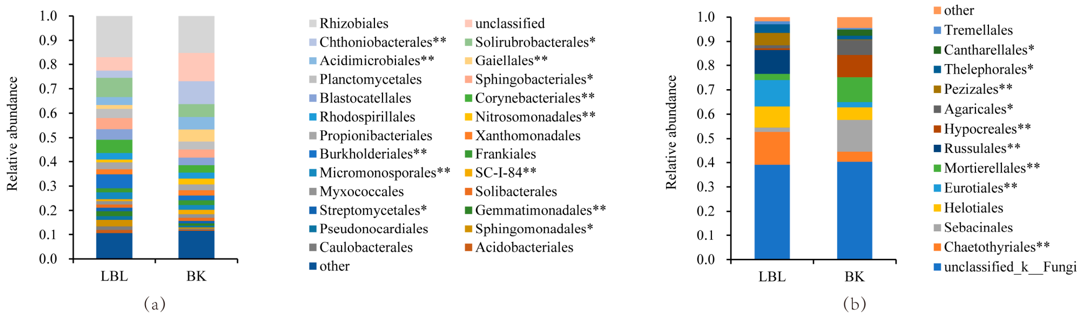

Figure S2. Bacterial (a) and fungal (b) community composition at the order. The order with a relative abundance of < 0.1% in soils were presented in other order". (** $p < 0.01$) and (* $p < 0.05$) indicate significant, differences between abundances in LBL and BK soils based on Wilcoxon signed-rank.

At the order level of bacterial community, the abundances of Sphingomonadales, Gemmatimonadales, Streptomycetales, Micromonosporales, Burkholderiales, Corynebacteriales, Sphingobacteriales and Solirubrobacterales were significantly higher in LBL than those in BK (Figure S2a). the abundances of SC-I-84, Nitrosomonadales, Gaiellales, Acidimicrobiales and Chthoniobacterales were significantly lower in LBL than those in BK (Figure S2a). At the order level of fungal community, the abundances of Chaetothyriales, Eurotiales, Russulales, Pezizales and Thelephorales were significantly higher in LBL than those in BK (Figure S2b). the abundances of Mortierellales, Hypocreales, Agaricales, and Cantharellales were significantly lower in LBL than those in BK (Figure S2b).

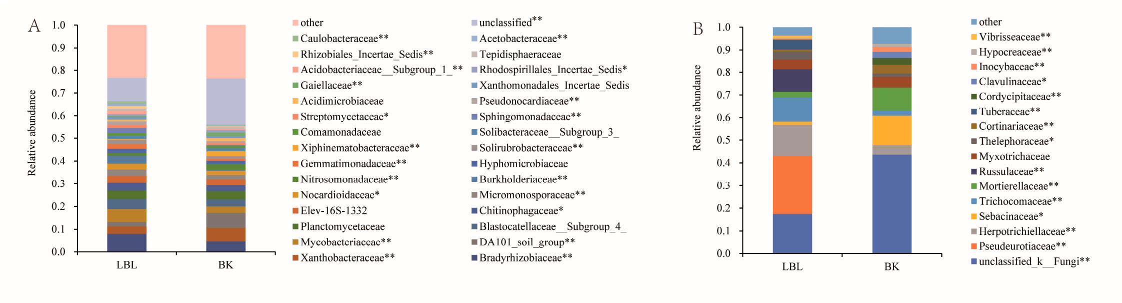

Figure S3. Bacterial (a) and fungal (b) community composition at the family. The family with a relative abundance of < 0.1% in soils were presented in other family". (** $p < 0.01$) and (* $p < 0.05$) indicate significant, differences between abundances in LBL and BK soils based on Wilcoxon signed-rank.

At the family level of bacterial community, the abundances of Bradyrhizobiaceae, Mycobacteriaceae, Chitinophagaceae, Micromonosporaceae, Nocardioidaceae, Burkholderiaceae, Gemmatimonadaceae, Solirubrobacteraceae, Sphingomonadaceae, Streptomycetaceae, Pseudonocardiaceae, Acidobacteriaceae-Subgroup-1, Rhizobiales-Incertae-Sedis, Acetobacteraceae and Caulobacteraceae were significantly higher in LBL than those in BK (Figure S3a). the abundances of Xanthobacteraceae, DA101-soil-group, Nitrosomonadaceae, Xiphinematobacteraceae, Gaiellaceae and Rhodospirillales-Incertae-Sedis were significantly lower in LBL than those in BK (Figure S3a). At the family level of fungal community, the abundances of Pseudeurotiaceae, Herpotrichiellaceae, Trichocomaceae, Russulaceae, Thelephoraceae, Tuberaceae and Vibrisseaceae were significantly in LBL higher than those in BK (Figure S3b). the abundances of Sebacinaceae, Mortierellaceae, Cortinariaceae, Cordycipitaceae, Clavulinaceae, Inocybaceae and Hypocreaceae were significantly lower in LBL than those in BK (Figure S3b).

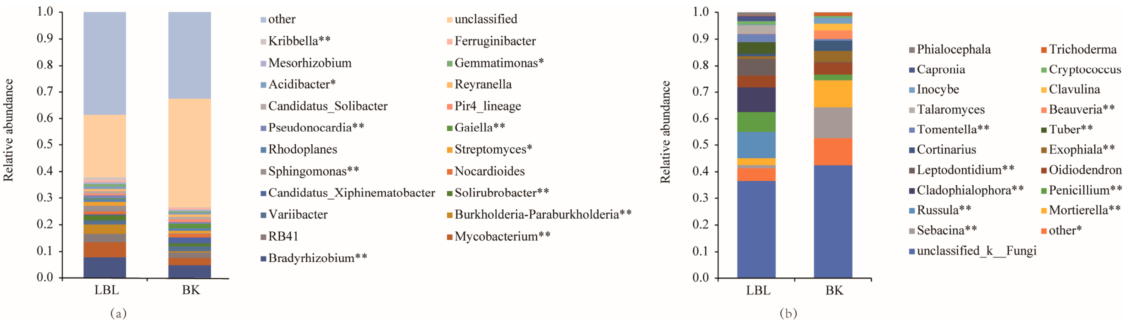

Figure S4. Bacterial (a) and fungal (b) community composition at the genus. The genus with a relative abundance of < 0.1% in soils were presented in other genus". (** $p < 0.01$) and (* $p < 0.05$) indicate significant, differences between abundances in LBL and BK soils based on Wilcoxon signed-rank.

At the genus level of bacterial community, the abundances of *Bradyrhizobium*, *Mycobacterium*, *Burkholderia-Paraburkholderia*, *Solirubrobacter*, *Sphingomonas*, *Streptomyces*, *Pseudonocardia*, *Acidibacter*, *Gemmatimonas* and *Kribbella* were significantly higher in LBL than those in BK (Figure S4a). the abundances of *Gaiella* was significantly lower in LBL than its in BK (Figure S4a). At the genus level of fungal community, the abundances of *Russula*, *Penicillium*, *Cladophialophora*, *Leptodontidium*, *Tuber* and *Tomentella* were significantly higher in LBL than those in BK (Figure S1b). the abundances of *Sebacina*, *Mortierella*, *Exophiala* and *Beauveria* were significantly lower in LBL than those in BK (Figure S1b).
